# Association of physical activity and screen time with cardiovascular disease risk in the Adolescent Brain Cognitive Development Study

**DOI:** 10.1186/s12889-024-18790-6

**Published:** 2024-05-18

**Authors:** Jason M. Nagata, Shayna Weinstein, Sana Alsamman, Christopher M. Lee, Erin E. Dooley, Kyle T. Ganson, Alexander Testa, Holly C. Gooding, Orsolya Kiss, Fiona C. Baker, Kelley Pettee Gabriel

**Affiliations:** 1 Department of Pediatrics, University of California, San Francisco, 550 16th Street, 4th Floor, Box 0503, San Francisco, CA 94143 USA; 2 Department of Epidemiology, University of Alabama at Birmingham, 1665 University Boulevard. Birmingham, Alabama, 35233 USA; 3 Factor-Inwentash Faculty of Social Work, University of Toronto, 246 Bloor St W, Toronto, ON M5S 1V4 Canada; 4 Department of Management, Policy and Community Health, University of Texas Health Science Center at Houston, 1200 Pressler Street, Houston, TX 77030 USA; 5 Division of General Pediatrics and Adolescent Medicine, Department of Pediatrics, Emory University School of Medicine, Children’s Healthcare of Atlanta, 2015 Uppergate Drive, Atlanta, GA 30322 USA; 6 Center for Health Sciences, SRI International, 333 Ravenswood Ave, Menlo Park, CA 94025 USA; 7 School of Physiology, University of the Witwatersrand, 1 Jan Smuts Ave, Braamfontein, Johannesburg, 2000 South Africa

**Keywords:** Digital media, Screen use, Physical activity, Adolescent, Cardiovascular disease, Blood pressure, Cholesterol, Diabetes, Dyslipidemia, Hemoglobin A1c, Hypertension

## Abstract

**Background:**

According to the Physical Activity Guidelines Advisory Committee Scientific Report, limited evidence is available on sedentary behaviors (screen time) and their joint associations with physical activity (steps) for cardiovascular health in adolescence. The objective of this study was to identify joint associations of screen time and physical activity categories with cardiovascular disease (CVD) risk factors (blood pressure, hemoglobin A1c, cholesterol) in adolescence.

**Methods:**

This study analyzed data from the Adolescent Brain Cognitive Development (ABCD) Study, comprising a diverse sample of 4,718 U.S. adolescents aged 10–15 years between 2018 and 2021. Steps were measured by a Fitbit wearable device and levels were categorized as low (1,000–6,000), medium (> 6,000–12,000), and high (> 12,000) averaged daily step counts. Self-reported recreational screen time hours per day were classified as low (0–4), medium (> 4–8), and high (> 8) hours per day. CVD risk factors including blood pressure, hemoglobin A1c, and cholesterol (total and HDL) were measured.

**Results:**

The analytical sample averaged 6.6 h of screen time per day and 9,722 steps per day. In models including both screen time and steps, the high screen time category was associated with a 4.27 higher diastolic blood pressure percentile (95% CI 1.83–6.73) and lower HDL cholesterol (B= -2.85, 95% CI -4.77 to -0.94 mg/dL) compared to the low screen time category. Medium (B = 3.68, 95% CI 1.24–6.11) and low (B = 7.64, 95% CI 4.07–11.20) step categories were associated with higher diastolic blood pressure percentile compared to the high step category. The medium step category was associated with lower HDL cholesterol (B= -1.99, 95% CI -3.80 to -0.19 mg/dL) compared to the high step category. Findings were similar when screen time and step counts were analyzed as continuous variables; higher continuous step count was additionally associated with lower total cholesterol (mg/dL).

**Conclusions:**

Combinations of low screen time and high steps were generally associated with favorable cardiovascular health markers including lower diastolic blood pressure and higher HDL cholesterol, which can inform future adolescent health guidelines.

**Supplementary Information:**

The online version contains supplementary material available at 10.1186/s12889-024-18790-6.

## Introduction

The U.S. Department of Health and Human Services’ *Physical Activity Guidelines for Americans* recommends 60 min of moderate-to-vigorous intensity physical activity per day for children and adolescents [1]. However, fewer than a quarter of U.S. adolescents met these guidelines prior to the COVID-19 pandemic [2, 3]. During the COVID-19 pandemic, physical activity among adolescents declined even further to 4.7% of adolescents meeting recommended physical activity levels [4]. This decline in physical activity has been accompanied by an increase in recreational screen time. In 2016, U.S. adolescents reported spending an average of 4–6 h of digital media engagement per day, including the internet, texting, and social media [5, 6], which doubled during the COVID-19 pandemic [7, 8].

Given limited discretionary hours in the day, an increase in screen time use displaces opportunities to be physically active [9]. This displacement is concerning, given emerging evidence supporting the harmful associations of sedentary behavior with cardiometabolic health and cardiovascular disease (CVD), which remains the leading cause of death in adults across most racial and ethnic groups in the U.S [10]. Adolescence is a critical period for CVD prevention because it is a time when healthy lifestyle behaviors can be established that will last into adulthood [11]. Higher screen time has been associated with higher CVD risk in children and adolescents, including higher blood pressure [12], non-HDL cholesterol [13], and insulin resistance [14, 15]. In contrast, physical activity has been associated with lower CVD risk, including an improved lipid profile, lower body fat, and lower blood pressure [16–18].

Despite findings that lower physical activity and greater screen time are generally associated with greater CVD risk, the Physical Activity Guidelines Advisory Committee Scientific Report identified important evidence gaps for research that could inform future guidelines. First, there was insufficient evidence to provide recommendations about sedentary behaviors, including screen time, during adolescence. Second, there is a lack of research on dose-response relationships between physical activity and cardiovascular health outcomes using objective measures. Finally, combinations of sedentary behavior and physical activity categories on cardiovascular health outcomes in adolescents should be further examined [18]. Understanding how various combinations of sedentary behavior and physical activity are associated with cardiovascular health outcomes in adolescents is crucial, as it can provide a more comprehensive insight into how lifestyle patterns contribute to the early development of cardiovascular risk factors, potentially informing more targeted and effective intervention strategies for this age group. For instance, adolescents who have long periods of sedentary time may require higher levels of physical activity to offset CVD risk [19].

Few studies have examined joint associations of screen time and physical activity on markers of cardiovascular health among adolescents. A 2016 Iranian study examined the association of screen time and physical activity on CVD risk in a nationally representative sample of school students and found a joint association of high screen time and low physical activity with higher odds of low HDL-cholesterol and elevated total cholesterol [20]. However, this study used a self-report measure of physical activity, which is limited by recall errors and response bias. Step count, in contrast, is an objective, device-recorded measure of physical activity that can be collected across a reasonably extended period of time [21]. Furthermore, step count provides estimates of accumulated daily movement using a metric that is meaningful to a lay audience, which may make them more clinically relevant. Additionally, combinations of physical activity and screen time have not been well studied among U.S. adolescents.

In this study, we address these gaps by examining the joint associations of physical activity (measured by daily steps) and sedentary behavior (screen time) exposures with CVD risk factors (blood pressure, hemoglobin A1c, and cholesterol levels). We hypothesize that high screen time and low steps would be associated with higher CVD risk in early adolescence.

## Methods

We examined cross-sectional data from Year 2 (2018–2020) and Year 3 (2019–2021) of the Adolescent Brain Cognitive Development (ABCD) Study (4.0 release; 2018–2020, ages 10–14 years), a longitudinal study of brain development and health in the U.S. In 2016–2018 (Year 0), 11,875 children were recruited from 21 demographically diverse sites distributed across the nation’s four major regions (Northeast, South, Midwest, West). Participants were recruited primarily through elementary schools, chosen via stratified, probability sampling of U.S. schools within the 21 catchment areas using the SAS V9.4. software system and the SAS Proc SurveySelect program. School selection was informed by gender, race/ethnicity, socioeconomic status, and urbanicity to minimize sample selection bias [22]. These sampling strategies aimed to maximize the representativeness of the baseline cohort with regards to the demographic and socioeconomic makeup of 9–10-year-old children in the U.S. While the sample is epidemiologically informed, self-selection by families into the study and assessment at academic centers may be a source of sampling bias. Response rates for individual students within schools and incompleteness at any particular school are not incorporated into the sample weighting schema. Further details regarding the study’s participants, recruitment process, procedures, and measures have been explained elsewhere [23, 24].

Because of the COVID-19 pandemic, Year 2 CVD risk measurement collection was disrupted due to social distancing requirements and the cancellation of non-essential research activities, and only a portion of the participants were able to complete certain CVD risk measurements. CVD risk measurements were re-attempted in Year 3 for some participants who were not able to have measurements in Year 2. Step count, screen time, and blood pressure data were collected and analyzed exclusively during Year 2 for all participants. Year 3 CVD outcome data for hemoglobin A1c and cholesterol was only utilized in the absence of data from Year 2. Overall, a majority (78.3%) of hemoglobin A1c and cholesterol data were collected in Year 2, while 21.7% were collected from Year 3. The current analysis included individuals with data for steps, screen time, and at least one CVD risk measurement (Additional File 1: Appendix A, B), resulting in a sample of 4,718 adolescents. The study was approved by the University of California, San Diego (UCSD) centralized Institutional Review Board (IRB), and the secondary data analysis was approved by the University of California, San Francisco (UCSF). Local IRBs from each study site also gave their approval. Caregivers/parents signed written informed consent forms prior to participation in the study. Adolescents signed written assent forms, given that they were minors, prior to participation in the study.

### Exposure variables

#### Screen time

Screen time data were collected using the ABCD Youth Screen Time Questionnaire, which asked participating adolescents to self-report the hours per day they typically spent using different types of media on weekdays and weekends [25]. Types of media included television shows, movies, videos, video chat, single and multi-player video games, social media, and texting. Adolescent-reported screen time has demonstrated a significant moderate positive correlation with an objectively sensed smartphone application among 11–12-year-old participants in the ABCD Study (*r* = 0.49, *p* < 0.001) [26]. Additional investigations have indicated that self-reported assessments of watching television were significantly moderately correlated (Spearman’s *p* = 0.54, *p* < 0.001) with an objectively recorded electronic television monitor and illustrated a high level of concurrence, with 95% of measurements falling within four hours of the average [27]. Comparable self-reported measures of television viewing have demonstrated satisfactory test-retest reliability (intraclass correlations over a seven-day period ranging from 0.76 to 0.81) [28, 29]. The time spent on all types of media was summed. A total weighted mean recreational screen use was calculated using the following weighting: ([weekday average x 5] + [weekend average x 2])/7. Screen time was calculated as a continuous variable and categorized into four-hour increments. This categorization was based on prior studies identifying four hours per day to be a threshold linked to poor mental health outcomes and overweight in adolescents [30–32], and other national surveys of adolescent screen time have used similar categories (e.g., 4 and 8 h per day) with similar distributions [19, 33]. Screen time (hours per day) was ordered into three categories: 0 to 4 h (low; reference category), 4 to 8 h (medium), and more than 8 h (high).

### Steps per day (Fitbit)

Daily step counts including weekdays, weekends, and holidays were collected through the Fitbit Charge (Fitbit Inc., San Francisco, CA) over a three-week period (21 days) between November 2018 and November 2020 that coincided with the Year 2 questionnaire and physical health assessments. Prior studies have shown Fitbit devices to be a reliable and accurate tool for the estimation of adolescents’ daily step counts to measure the accumulated physical activity in adolescents over time [21, 34]. We observed best practices for data extraction, filtering, and processing established by the ABCD Study [21, 34]. Following earlier studies, we incorporated all days with > 599 daily minutes of wear time while awake and a minimum of 1,000 steps per day within each adolescent’s three-week study protocol [35–39]. In our ABCD Study Fitbit data, 1,000 steps per day represented the bottom 0.5th percentile (2.58 standard deviations below the mean), consistent with normative ranges published for step counts among 10-11-year-olds from the National Health and Nutrition Examination Surveys [40]. Prior research identified 12,000 steps as a lower threshold for satisfying the 60 min of moderate-to-vigorous intensity physical activity guideline for adolescents from the Department of Health and Human Services’ *Physical Activity Guidelines for Americans* [41]. Accordingly, 6,000 steps approximate 30 min of moderate-to-vigorous intensity physical activity, or half the adolescent guideline threshold. Therefore, total steps per day were classified into three categories: 1,000 to 6,000 steps per day (low), 6,000 to 12,000 steps per day (medium), and more than 12,000 steps per day (high, reference category).

### Outcome variables

#### Cardiovascular disease risk factors

Continuous measures were considered primary outcomes. Given the low prevalence of clinical cutoffs and subsequently less power, binary clinical outcomes were considered secondary outcomes.

### Blood pressure percentile

ABCD Study research assistants were trained on the standardized protocol used at all sites. Prior to measurement, participants sat in a chair for 5 min in a quiet environment. The participant’s right arm was rested palm face up on a table, and feet were positioned flat on the floor, legs uncrossed. Blood pressure was calculated using the mean of three measurements separated by a 60 s interval using a factory-calibrated, Omron blood pressure monitor (MicroLife USA, Inc.; Dunedin, FL). Cuff size was determined by measurement of the mid-upper arm circumference. Systolic and diastolic blood pressures were converted into percentiles based on the American Academy of Pediatrics reference ranges [42]. Hypertensive range blood pressure (secondary outcome) was defined as appropriate for age and sex percentile according to pediatric guidelines for elevated blood pressure [42]. Participants taking antihypertensive medications (*n* = 2) were excluded from the analyses with blood pressure as an outcome.

### Hemoglobin A1c

Hemoglobin A1c level was measured via blood draw as a measure of average blood sugar levels over the prior three months [43]. Participants were determined to have testing consistent with diabetes (secondary outcome) if they had a hemoglobin A1c level ≥ 6.5% [43]. Adolescent participants with a parent-reported history of diabetes (*n* = 15) were excluded from the analyses with hemoglobin A1c or diabetes as an outcome.

### Cholesterol

Non-fasting total cholesterol and High-Density Lipoprotein (HDL) cholesterol were collected via blood draw. Hyperlipidemia (secondary outcome) was defined as total cholesterol ≥ 200 mg/dL [44]. Low HDL cholesterol (secondary outcome) was defined as < 40 mg/dL for female and male adolescents [44].

### Covariates

We included as covariates parent-reported measures of marital status, highest parent education, household income, adolescent age, adolescent race/ethnicity (White, Latinx/Hispanic, Black, Asian, Native American, other), and adolescent sex (female or male), which have been previously linked to adolescent physical activity [45], screen time use [6], and CVD risk [46]. We constructed a COVID-19 pandemic variable using Fitbit device data collection dates (before, before and during, and during the COVID-19 pandemic), with March 13, 2020 as the start of the COVID-19 pandemic in the U.S, when a national emergency was declared. Because the Fitbit data were collected over 21 days, there was a small subgroup for whom the 21-day period started before March 13, 2020 but ended after March 13, 2020. These participants were considered “before and during the COVID-19 pandemic.” We additionally adjusted for calendar month as a proxy for seasonality given that seasonality could affect screen time and physical activity [47]. We also adjusted for study year in the analysis of hemoglobin A1c, total cholesterol, and HDL cholesterol given that those measures were collected across Years 2 and 3.

### Statistical analysis

Data analysis was performed using Stata software, version 18 (StataCorp LLC). Descriptive statistics were calculated including means, standard deviations, and percentages. Multivariable linear regression analyses were conducted to estimate associations between exposure variables (screen time and steps, continuous and categorical variables) and continuous CVD risk factor outcomes (primary outcomes: systolic and diastolic blood pressure percentiles, hemoglobin A1c, and total and HDL cholesterol). Multivariable logistic regression analyses were conducted to estimate associations between exposure variables (screen time and steps categories) and binary CVD risk factor outcomes (secondary outcomes: hypertensive range blood pressure, testing consistent with diabetes, high total cholesterol, low HDL cholesterol). All models adjusted for age, sex, race/ethnicity, household income, parental education, parent marital status, data collection period (e.g., before, before and during, and during the COVID-19 pandemic), month, and study year (e.g., Year 2 or Year 3 for the collection of the CVD outcome). For each outcome with significant associations with both screen time and step categories, we reported a 9-category exposure (combinations of 3 screen time categories and 3 step count categories) to estimate the association of each screen-step category combination with the CVD risk factor. We assessed for effect modification (interactions) between screen time and step categories for the association with each CVD risk factor outcome. We also assessed for effect modification (interactions) by race/ethnicity for the associations between screen time and steps with each CVD risk factor outcome.

## Results

A total of 4,718 adolescents were included in this analysis. Overall, 47.6% of the participants were female and 44.7% were racial/ethnic minorities, with a mean age of 12.0 years. Adolescents reported an average of 6.6 h of screen time per day. The average daily step count calculated across the Fitbit wear period was 9722.2 steps per day (Table 1).

**Table 1:** Sample characteristics of Adolescent Brain Cognitive Development (ABCD) Study participants included in the current analysis (*N* = 4,718)

Sociodemographic characteristics	Mean (SD)
Age at the time of screen time and Fitbit data collection (years)	12.0 (0.7)
Sex, n (%)	
Female	2,244 (47.6%)
Male	2,474 (52.4%)
Race/ethnicity, n (%)	
White	2,609 (55.3%)
Latino / Hispanic	726 (15.4%)
Black	854 (18.1%)
Asian	267 (5.7%)
Native American	206 (4.4%)
Other	56 (1.2%)
Household income, n (%)	
Less than $75,000	1,600 (36.5%)
75,000 or more	2,782 (63.5%)
Parent education, n (%)	
High school education or less	605 (12.9%)
Some college education or more	4,104 (87.2%)
Parent marital status, n (%)	
Parent married/partnered	3,547 (75.7%)
Parent not married/unpartnered	1,137 (24.3%)
Physical activity variables (step count)	
Total steps per day	9722.2 (3346.7)
Step categories, n (%)	
1,000 to 6,000 steps per day	654 (18.4%)
> 6,000 to 12,000 steps per day	2,440 (68.5%)
> 12,000 steps per day	468 (13.1%)
Screen time (hours)	
Total recreational screen time	6.6 (5.6)
Screen time categories, n (%)	
0 to 4 h per day	1,962 (41.7%)
> 4 to 8 h per day	1,446 (30.8%)
> 8 h per day	1,293 (27.5%)
Cardiovascular disease risk measures	
Systolic blood pressure percentile (*n* = 4,042)	41.7 (29.2)
Diastolic blood pressure percentile (*n* = 4,042)	43.4 (24.2)
Hemoglobin A1c (percent, *n* = 1,588)	5.2 (0.3)
Total cholesterol (mg/dL, *n* = 1,540)	156.9 (28.8)
HDL cholesterol (mg/dL, *n* = 1,538)	56.0 (12.8)
Data collection period, n (%)	
Before the COVID-19 pandemic^a^	2,886 (81.9%)
Before and during the COVID-19 pandemic^b^	183 (5.2%)
During the COVID-19 pandemic^c^	453 (12.9%)

SD = standard deviation. Cells may not add up to 4,718 due to missing data

^a^ All physical activity data collected before March 13, 2020

^b^ Physical activity data collection started before March 13, 2020 but ended on or after March 13, 2020

^c^ All physical activity data collected on or after March 13, 2020

### Blood pressure percentile

**Fig. 1:**
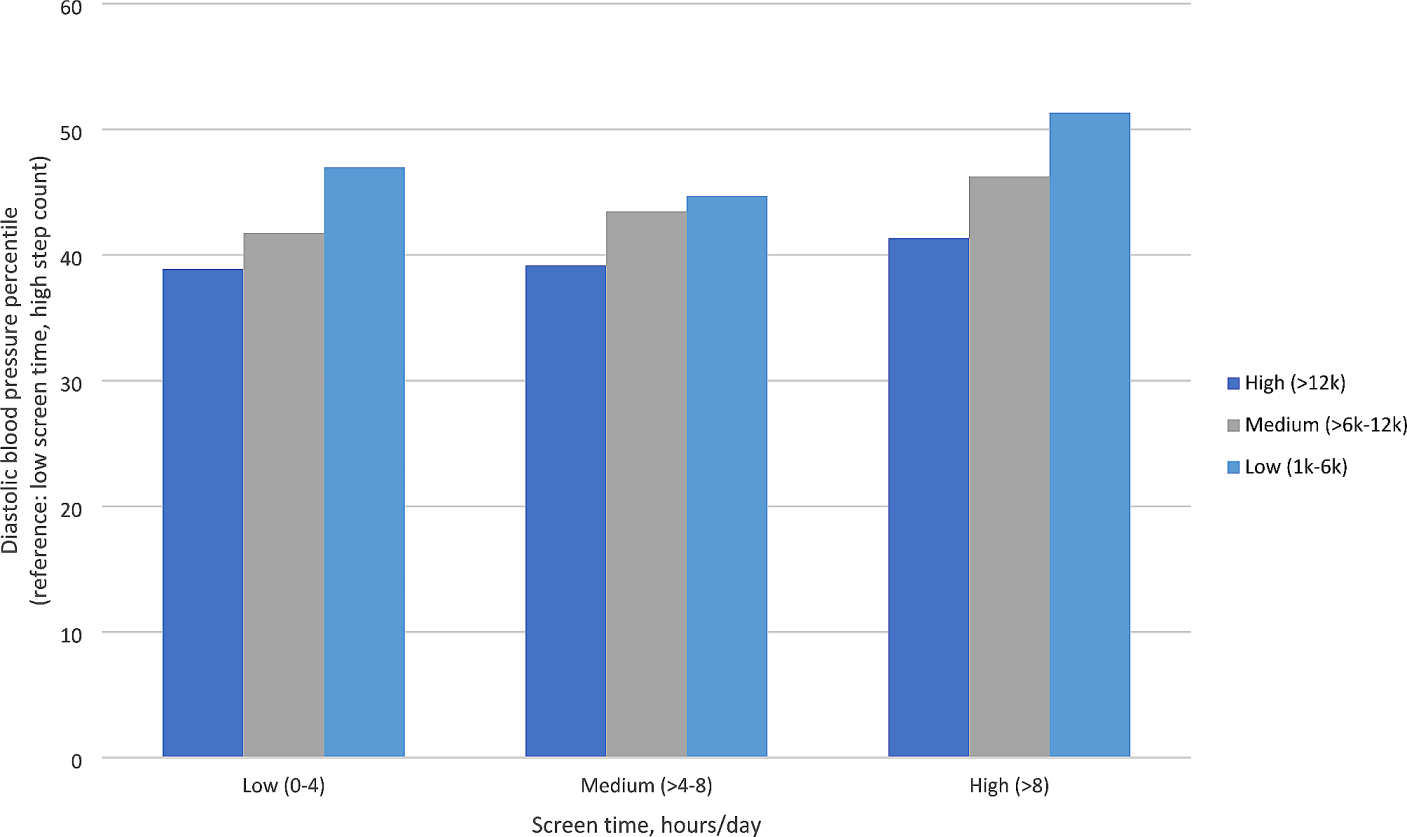
Associations between screen time and step count category combinations and diastolic blood pressure percentile. Legend: Results correspond to coefficients from a linear regression model with nine categories of screen time and step combinations as the independent variable and diastolic blood pressure percentile as the dependent variable, adjusting for age, sex, race/ethnicity, household income, parental education, parent marital status, data collection period, and month. Daily step categories included: high (> 12,000), medium (6,000–12,000), and low (1,000–6,000). Daily screen time categories (hours) included: low (0–4); medium (4–8), high (> 8). The low screen time and high step category was the reference category

In linear regression models including both screen and steps as continuous variables (Table 2), each hour of screen time per day was associated with a 0.27 (95% CI 0.06 to 0.48) higher diastolic blood pressure percentile, and every 1,000 steps per day was associated with a 0.66 (95% CI 0.32 to 0.99) lower diastolic blood pressure percentile. When examining categories, the high screen time category was associated with a 4.27 higher diastolic blood pressure percentile (95% CI 1.83 to 6.73) compared to the low screen time category. The medium step category was associated with a 3.68 (95% CI 1.24 to 6.11) higher diastolic blood pressure percentile, and the low step category was associated with a 7.64 (95% CI 4.07 to 11.20) higher diastolic blood pressure percentile, compared to the high step category. We further examined combinations of the 3 screen and 3 step categories (9 categories total) and diastolic blood pressure percentile (Fig. 1). For each screen time category, low step count categories were associated with higher diastolic blood pressure percentile. There were no significant associations among screen time or steps (continuous variables and categories) and systolic blood pressure percentile (Table 2).

**Table 2:** Associations between screen time and step count categories and cardiovascular disease risk (CVD) outcomes in Adolescent Brain Cognitive Development (ABCD) Study participants included in the current analysis

Screen time (hrs/day)	B (95% CI)	*p*	Steps/day	B (95% CI)	*p*
Systolic blood pressure percentile					
Continuous	0.13 (-0.13 to 0.38)	0.326	Continuous	0.35 (-0.06 to 0.76)	0.096
Categories			Categories		
Low (0–4)	Reference		High (> 12,000)	Reference	
Medium (> 4–8)	0.51 (-2.05 to 3.08)	0.695	Medium (> 6,000–12,000)	-1.72 (-4.70 to 1.25)	0.256
High (> 8)	2.72 (-0.27 to 5.71)	0.075	Low (1,000–6,000)	-3.67 (-8.02 to 0.68)	0.098
Diastolic blood pressure percentile					
Continuous	**0.27 (0.06 to 0.48)**	**0.011**	Continuous	**-0.66 (-0.99 to -0.32)**	**< 0.001**
Categories			Categories		
Low (0–4)	Reference		High (> 12,000)	Reference	
Medium (> 4–8)	1.01 (-1.10 to 3.11)	0.348	Medium (> 6,000–12,000)	**3.68 (1.24 to 6.11)**	**0.003**
High (> 8)	**4.27 (1.83 to 6.73)**	**0.001**	Low (1,000–6,000)	**7.64 (4.07 to 11.20)**	**< 0.001**
Hemoglobin A1c					
Continuous	0.00 (0.00 to 0.01)	0.795	Continuous	0.00 (0.00 to 0.01)	0.918
Categories			Categories		
Low (0–4)	Reference		High (> 12,000)	Reference	
Medium (> 4–8)	0.04 (-0.01, 0.09)	0.113	Medium (> 6,000–12,000)	0.01 (-0.05, 0.06)	0.774
High (> 8)	0.01 (-0.05, 0.07)	0.695	Low (1,000–6,000)	0.01 (-0.08, 0.09)	0.855
Total Cholesterol					
Continuous	-0.22 (-0.55 to 0.12)	0.199	Continuous	**-0.58 (-1.12 to -0.04)**	**0.035**
Categories			Categories		
Low (0–4)	Reference		High (> 12,000)	Reference	
Medium (> 4–8)	-0.79 (-4.62, 3.04)	0.686	Medium (> 6,000–12,000)	**5.12 (1.09, 9.16)**	**0.013**
High (> 8)	-2.77 (-7.05 to 1.52)	0.206	Low (1,000–6,000)	4.69 (-1.86, 11.25)	0.161
HDL cholesterol					
Continuous	**-0.18 (-0.33 to -0.03)**	**0.020**	Continuous	0.22 (-0.03 to 0.46)	0.080
Categories			Categories		
Low (0–4)	Reference		High (> 12,000)	Reference	
Medium (> 4–8)	-1.32 (-3.03 to 0.39)	0.131	Medium (> 6,000–12,000)	**-1.99 (-3.80 to -0.19)**	**0.030**
High (> 8)	**-2.85 (-4.77 to -0.94)**	**0.004**	Low (1,000–6,000)	-2.14 (-5.07 to 0.79)	0.153

All models include screen time and physical activity (step count) as the joint independent variables and were adjusted for age, sex, race/ethnicity, household income, parental educational level, parental marital status, data collection period (i.e., before the COVID-19 pandemic, before and during the COVID-19 pandemic, or during the COVID-19 pandemic), and calendar month. The continuous step count is for 1,000 steps per day. For hemoglobin A1c, total cholesterol, and HDL cholesterol, time between independent and dependent variable was also adjusted for given that some measures were collected across Years 2 and 3. Participants with a prior diagnosis of diabetes were excluded from the analysis of hemoglobin A1c and participants on hypertension medications were excluded from analyses of systolic and diastolic blood pressure

### Total cholesterol and HDL cholesterol

**Fig. 2:**
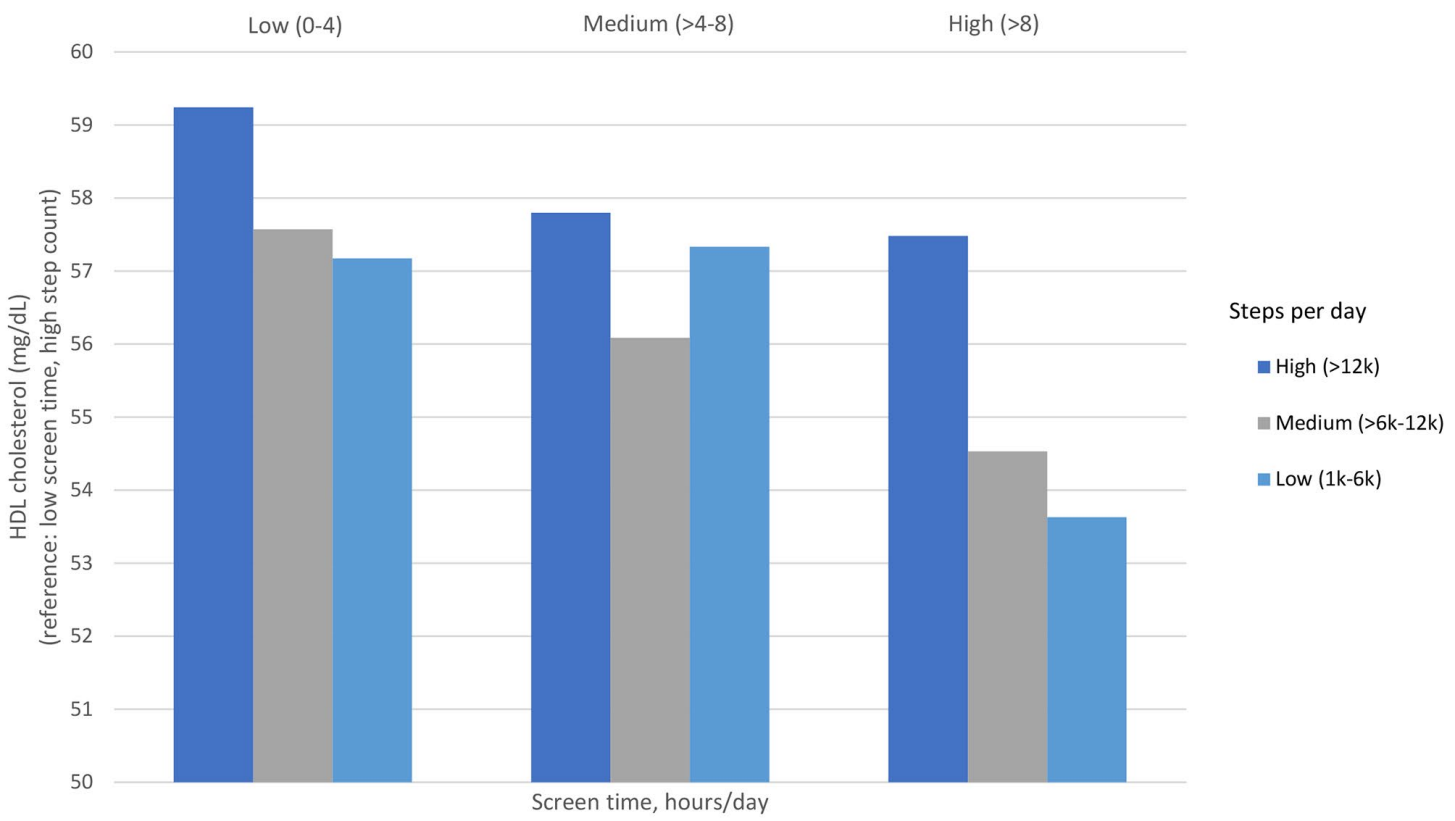
Associations between screen time and step count category combinations and HDL cholesterol. Legend: Results correspond to coefficients from a linear regression model with nine categories of screen time and step combinations as the independent variable and HDL cholesterol as the dependent variable, adjusting for age, sex, race/ethnicity, household income, parental education, parent marital status, data collection period, month, and study year. Daily step categories included: high (> 12,000), medium (6,000–12,000), and low (1,000–6,000). Daily screen time categories (hours) included: low (0–4); medium (4–8), high (> 8). The low screen time and high step category was the reference category

In linear regression models including both screen and steps as continuous variables, each hour of screen time per day was associated with a 0.18 (95% CI 0.03 to 0.33) mg/dL lower HDL cholesterol; however, steps per day were not significantly associated with HDL cholesterol. When examining categories, the medium step category was associated with − 1.99 mg/dL lower HDL cholesterol (95% CI -3.80 to -0.19) compared to the high step category. High screen time was associated with − 2.85 mg/dL lower HDL cholesterol (95% CI -4.77 to − 0.94) compared to the low screen time category. We further examined combinations of the 3 screen and 3 step categories (9 categories total) and HDL cholesterol (Fig. 2). For participants in the low screen time category, low and medium steps were associated with lower HDL cholesterol compared to participants in the high step category.

For total cholesterol, every 1,000 steps per day was associated with a 0.58 (95% CI 0.04 to 1.12) lower total cholesterol, and screen time was not significantly associated with total cholesterol (B = -0.22, 95% CI -0.55 to 0.12). When examining categories, the medium step category was associated with higher total cholesterol compared to the high step category (B = 5.12, 95% CI 1.09 to 9.16). Screen time categories were not significantly associated with total cholesterol (Table 2).

### Hemoglobin A1c

There were no significant associations between screen time or steps and hemoglobin A1c (Table 2).

Sensitivity analyses with logistic regression models including both screen and step categories and binary outcomes are shown in Additional File 1: Appendix B. There was no evidence of significant interactions between screen time and step categories for each of the CVD risk outcomes (all p for interaction > 0.05). There was no evidence of significant effect modification by race/ethnicity for the associations between screen time and steps for each of the CVD risk outcomes (all p for interaction > 0.05).

## Discussion

In this analysis of a national, demographically diverse sample of 10-15-year-old adolescents in the ABCD Study in the U.S., several gaps identified in the 2018 *Physical Activity Guidelines for Americans* are addressed [1, 18]. First, we found that high recreational screen time was associated with higher diastolic blood pressure percentile and lower HDL cholesterol, even when accounting for physical activity. Second, we found a dose-response relationship between step count categories and diastolic blood pressure percentile, with the lowest step category being associated with the highest diastolic blood pressure percentile. Third, we did not find evidence of effect modification between screen time and physical activity for diastolic blood pressure percentile and HDL cholesterol. The use of categories and continuous screen time and steps variables, as well as considering joint associations between screen time and steps on CVD risk, can inform gaps in public health and clinical guidance for adolescents.

Daily screen time of more than 8 h was associated with higher diastolic blood pressure percentile, even when adjusting for daily steps. Screen time is mostly a sedentary behavior, which displaces physical activity and can lead to an increase in caloric consumption through mechanisms such as mindless snacking and advertisements that promote unhealthy foods [48, 49]. We have previously shown that greater screen time is associated with higher BMI percentile in the ABCD Study [19, 50]. In addition, contemporary screen modalities (e.g., social media, video games) may lead to exposure to cyberbullying, violence, or other stressful content that could raise blood pressure [32, 51]. These mechanisms may explain why high screen time may be associated with poorer cardiovascular health. The study builds on prior literature by incorporating several contemporary modalities in screen time, adjusting for step counts, and focusing on early adolescence which is an important developmental period for the development of lifestyle behaviors that can persist into adulthood, affecting cardiovascular health across the lifespan.

On average, adolescents recorded 9,722 daily steps, which is consistent with estimates from the National Health and Nutrition Examination Survey for 10-11-year-old adolescents [40] and other smaller studies with similar age groups [52, 53]. This average is below the 12,000 steps per day threshold which approximates the recommended 60 min of moderate-to-vigorous intensity physical activity per day for adolescents from the *Physical Activity Guidelines for Americans* [41]. When examining the independent associations of step count on cardiovascular outcomes, we found a dose-response relationship between lower step count categories and higher diastolic blood pressure percentile. Previous studies have demonstrated that engaging in physical activity throughout adolescence is associated with a lower risk of hypertension, suggesting that exercise may have a protective effect on blood pressure. We identified one study that utilized step count as a summary estimate of physical activity, demonstrating a negative correlation between step count and risk of hypertension in children and adolescents [16]. Our study found that fewer steps was associated with higher diastolic blood pressure percentile, with no statistically significant association found for systolic blood pressure percentile. Importantly, previous studies have demonstrated that diastolic blood pressure (compared to systolic blood pressure) is a stronger predictor of CVD risk in adolescents [54, 55].

In addition, having a higher daily step count was associated with a higher HDL cholesterol. Previous studies have shown that self-reported physical activity is associated with an improved lipid profile [56–58]. Our findings add to prior literature by indicating that even after accounting for screen time, a higher step count as a measure of physical activity is specifically associated with higher HDL cholesterol.

Evidence of effect modification between physical activity and screen time was not observed for diastolic blood pressure percentile and HDL cholesterol. For each screen time category, low step count categories were associated with higher diastolic blood pressure percentile and lower HDL cholesterol. Overall, our findings are consistent with previous studies which have reported a joint association between high screen time and low physical activity on CVD risk [20, 59], but extend prior findings by expanding screen time to include contemporary modalities (e.g., social media, texting, video chat, video games) and by using step count via devices as an objective measure of physical activity in early adolescents.

Limitations of this study include its cross-sectional nature. Fitbit device data were collected for a 3-week period (21 days), which may not be representative of a participant’s physical activity or the intensity of activity over the course of one year. Future studies may utilize longitudinal Fitbit data for longer durations to overcome this limitation, as well as to explore potential seasonal differences. While only days with > 599 min of waking wear were included in the analysis, variations in wear time above this threshold were not controlled for, thus producing a potential limitation with regards to differences in participants’ total wear time and variations in wake vs. sleep time. Fitbit devices may miss some activity data (e.g., biking, skateboarding), as it is mainly worn on the wrist. It is possible that wearing activity monitors like Fitbit could increase adolescent physical activity given real-time feedback regarding activity level; however, one prior study did not find that wearing a Fitbit increased physical activity levels in 10-year-olds [52]. With regards to screen time, measures were based on self-reported data, which are subject to recall errors and reporting bias. Additionally, the measurement of screen time did not account for the content and intensity of engagement. There is the potential for unmeasured confounders, although we controlled for site and sociodemographic factors as well as the COVID-19 pandemic, study year, and seasonality. We did not control for adiposity as it was not directly measured in the ABCD Study and adiposity could be a mediator in addition to a confounder for the association among screen time, steps, and CVD risk factor outcomes [58]. While the sample is epidemiologically informed, self-selection by families into the study and assessment at academic centers may be a source of sampling bias. Due to the COVID-19 pandemic, there was missing data which could lead to selection bias as participants included were more likely to be White, have a household income $75,000 or more, and have married/partnered parents (Appendix B). For a minority of participants (21.7%), blood draws (e.g., hemoglobin A1c and cholesterol) were measured one year later than the screen time and Fitbit measures; however, this would have been during a similar month/season and we controlled for data collection year in the analysis.

The strengths of this study include the socio-demographically diverse and large population-based sample, the use of objective data over 21 days (longer than a more typical 7-day protocol) which limits self-report bias and decreases standard error for physical activity measures, and including several different types of screen mediums used by adolescents rather than just computer and television for the screen time measure.

## Conclusion

The study adds to the literature by addressing evidence gaps identified by the Physical Activity Guidelines Advisory Committee Scientific Report by identifying specific categories of screen time and step count associated with CVD risk in early adolescence. In our study, more than 8 h of daily screen time and less than 12,000 steps per day were associated with higher diastolic blood pressure percentile among a racially diverse U.S. adolescent population-based sample. More than 8 h of daily screen time was also associated with lower HDL cholesterol. Future research should use a longitudinal study design and analyze differences by weekdays, weekends, or holidays, which would further inform physical activity and screen time guidelines for adolescents.

### Electronic supplementary material

Below is the link to the electronic supplementary material.

Additional File 1: Appendix A. Flow diagram of included participants. Appendix B. Comparison of participants included vs excluded due to missing data; Appendix C. Associations between screen time and step count categories and binary cardiovascular disease risk (CVD) outcomes in the Adolescent Brain Cognitive Development (ABCD) Study

## Data Availability

Data used in the preparation of this article were obtained from the ABCD Study (https://abcdstudy.org), held in the NIMH Data Archive (NDA).

## Abbreviations

ABCD: Adolescent Brain Cognitive Development Study
CVD: Cardiovascular disease
UCSD: University of California, San Diego
UCSF: University of California, San Francisco

## References

[1] U.S. Department of Health and Human Services (2018). Physical activity guidelines for americans.

[2] Katzmarzyk PT, Denstel KD, Beals K, Carlson J, Crouter SE, McKenzie TL (2018). Results from the United States 2018 Report Card on Physical Activity for Children and Youth. J Phys Act Health.

[3] Nagata JM, Cortez CA, Dooley EE, Iyer P, Ganson KT, Pettee Gabriel K (2022). Moderate-to-vigorous intensity physical activity among adolescents in the USA during the COVID-19 pandemic. Prev Med Rep.

[4] Cortez CA, Yuefan Shao I, Seamans MJ, Dooley EE, Pettee Gabriel K, Nagata JM (2023). Moderate-to-vigorous intensity physical activity among U.S. adolescents before and during the COVID-19 pandemic: findings from the adolescent brain Cognitive Development Study. Prev Med Rep.

[5] Twenge JM, Martin GN, Spitzberg BH (2019). Trends in U.S. adolescents’ media use, 1976–2016: the rise of digital media, the decline of TV, and the (near) demise of print. Psychol Popular Media Cult.

[6] Nagata JM, Ganson KT, Iyer P, Chu J, Baker FC, Pettee Gabriel K (2022). Sociodemographic correlates of contemporary screen Time Use among 9- and 10-Year-old children. J Pediatr.

[7] Nagata JM, Cortez CA, Cattle CJ, Ganson KT, Iyer P, Bibbins-Domingo K (2022). Screen time use among U.S. adolescents during the COVID-19 pandemic: findings from the adolescent brain Cognitive Development (ABCD) study. JAMA Pediatr.

[8] Kiss O, Nagata JM, de Zambotti M, Dick AS, Marshall AT, Sowell ER (2023). Effects of the COVID-19 pandemic on screen time and sleep in early adolescents. Health Psychol.

[9] Sandercock GRH, Ogunleye A, Voss C (2012). Screen time and physical activity in youth: thief of time or lifestyle choice?. J Phys Act Health.

[10] Tsao CW, Aday AW, Almarzooq ZI, Anderson CAM, Arora P, Avery CL (2023). Heart Disease and Stroke Statistics-2023 update: a Report from the American Heart Association. Circulation.

[11] Chung RJ, Touloumtzis C, Gooding H (2015). Staying Young at Heart: Cardiovascular Disease Prevention in adolescents and Young adults. Curr Treat Options Cardiovasc Med.

[12] Cassidy-Bushrow AE, Johnson DA, Peters RM, Burmeister C, Joseph CLM (2015). Time spent on the internet and adolescent blood pressure. J Sch Nurs.

[13] Sivanesan H, Vanderloo LM, Keown-Stoneman CDG, Parkin PC, Maguire JL, Birken CS (2020). The association between screen time and cardiometabolic risk in young children. Int J Behav Nutr Phys Act.

[14] Nightingale CM, Rudnicka AR, Donin AS, Sattar N, Cook DG, Whincup PH (2017). Screen time is associated with adiposity and insulin resistance in children. Arch Dis Child.

[15] Nagata JM, Lee CM, Lin F, Ganson KT, Pettee Gabriel K, Testa A (2023). Screen time from adolescence to Adulthood and Cardiometabolic Disease: a prospective cohort study. J Gen Intern Med.

[16] Weres A, Baran J, Czenczek-Lewandowska E, Leszczak J, Mazur A (2022). The association between steps per day and blood pressure in children. Sci Rep.

[17] Ramires VV, Dumith SC, Gonçalves H (2015). Longitudinal Association between Physical Activity and Body Fat during Adolescence: a systematic review. J Phys Act Health.

[18] 2018 Physical Activity Guidelines Advisory Committee. 2018 Physical Activity Guidelines Advisory Committee Scientific Report. Washington, DC: U.S. Department of Health and Human Services; 2018.

[19] Nagata JM, Smith N, Alsamman S, Lee CM, Dooley EE, Kiss O (2023). Association of physical activity and screen time with body Mass Index among US adolescents. JAMA Netw Open.

[20] Heshmat R, Qorbani M, Babaki AES, Djalalinia S, Ataei-Jafari A, Motlagh ME (2016). Joint Association of Screen Time and physical activity with Cardiometabolic Risk Factors in a National Sample of Iranian adolescents: the CASPIANIII Study. PLoS ONE.

[21] Bagot KS, Matthews SA, Mason M, Squeglia LM, Fowler J, Gray K (2018). Current, future and potential use of mobile and wearable technologies and social media data in the ABCD study to increase understanding of contributors to child health. Dev Cogn Neurosci.

[22] Compton WM, Dowling GJ, Garavan H (2019). Ensuring the best use of data: the adolescent brain Cognitive Development Study. JAMA Pediatr.

[23] Barch DM, Albaugh MD, Avenevoli S, Chang L, Clark DB, Glantz MD (2018). Demographic, physical and mental health assessments in the adolescent brain and cognitive development study: rationale and description. Dev Cogn Neurosci.

[24] Garavan H, Bartsch H, Conway K, Decastro A, Goldstein RZ, Heeringa S (2018). Recruiting the ABCD sample: design considerations and procedures. Dev Cogn Neurosci.

[25] Bagot KS, Tomko RL, Marshall AT, Hermann J, Cummins K, Ksinan A (2022). Youth screen use in the ABCD® study. Dev Cogn Neurosci.

[26] Wade NE, Ortigara JM, Sullivan RM, Tomko RL, Breslin FJ, Baker FC (2021). Passive sensing of preteens’ smartphone use: an adolescent brain Cognitive Development (ABCD) cohort substudy. JMIR Ment Health.

[27] Otten JJ, Littenberg B, Harvey-Berino JR (2010). Relationship between self-report and an objective measure of television-viewing time in adults. Obes (Silver Spring).

[28] Pettee KK, Ham SA, Macera CA, Ainsworth BE (2009). The reliability of a survey question on television viewing and associations with health risk factors in US adults. Obes (Silver Spring).

[29] Vereecken CA, Todd J, Roberts C, Mulvihill C, Maes L (2006). Television viewing behaviour and associations with food habits in different countries. Public Health Nutr.

[30] Hume C, Singh A, Brug J, van Mechelen W, Chinapaw M (2009). Dose-response associations between screen time and overweight among youth. Int J Pediatr Obes.

[31] Zink J, Belcher BR, Kechter A, Stone MD, Leventhal AM (2019). Reciprocal associations between screen time and emotional disorder symptoms during adolescence. Prev Med Rep.

[32] Nagata JM, Chu J, Ganson KT, Murray SB, Iyer P, Gabriel KP (2022). Contemporary screen time modalities and disruptive behavior disorders in children: a prospective cohort study. J Child Psychol Psychiatry.

[33] Rideout V, Robb M. The common sense census: media use by tweens and teens. Common Sense Media. 2019;1–104.

[34] Godino JG, Wing D, de Zambotti M, Baker FC, Bagot K, Inkelis S et al. Performance of a commercial multi-sensor wearable (Fitbit Charge HR) in measuring physical activity and sleep in healthy children. PLoS ONE. 2020;15. 10.1371/journal.pone.0237719 PMC7473549 32886714

[35] St Fleur RG, St George SM, Leite R, Kobayashi M, Agosto Y, Jake-Schoffman DE (2021). Use of Fitbit devices in physical activity intervention studies across the Life Course: Narrative Review. JMIR Mhealth Uhealth.

[36] van Woudenberg TJ, Bevelander KE, Burk WJ, Smit CR, Buijs L, Buijzen M (2018). A randomized controlled trial testing a social network intervention to promote physical activity among adolescents. BMC Public Health.

[37] Hemphill NM, Kuan MTY, Harris KC (2020). Reduced physical activity during COVID-19 pandemic in children with congenital heart disease. Can J Cardiol.

[38] Hoeger WWK, Bond L, Ransdell L, Shimon JM, Merugu S (2008). One-mile step count at walking and running speeds. ACSM’s Health Fit J.

[39] Lubans DR, Plotnikoff RC, Miller A, Scott JJ, Thompson D, Tudor-Locke C (2015). Using pedometers for measuring and increasing physical activity in children and adolescents: the next step. Am J Lifestyle Med.

[40] Barreira TV, Schuna JM, Mire EF, Broyles ST, Katzmarzyk PT, Johnson WD (2015). Normative Steps/Day and peak cadence values for United States Children and adolescents: National Health and Nutrition Examination Survey 2005–2006. J Pediatr.

[41] Colley RC, Janssen I, Tremblay MS (2012). Daily step target to measure adherence to physical activity guidelines in children. Med Sci Sports Exerc.

[42] Flynn JT, Kaelber DC, Baker-Smith CM, Blowey D, Carroll AE, Daniels SR (2017). Clinical practice Guideline for Screening and Management of High Blood pressure in children and adolescents. Pediatrics.

[43] Arslanian S, Bacha F, Grey M, Marcus MD, White NH, Zeitler P (2018). Evaluation and management of Youth-Onset type 2 diabetes: a position Statement by the American Diabetes Association. Diabetes Care.

[44] National Heart, Lung and Blood Institute. Expert Panel on Integrated Guidelines for Cardiovascular Health and Risk Reduction in Children and Adolescents: Summary Report. In: Pediatric Clinical Practice Guidelines & Policies. 14th edition. American Academy of Pediatrics. 2014. pp. 1099–1099. 10.1542/peds.2009-2107C PMC4536582 22084329

[45] Armstrong S, Wong CA, Perrin E, Page S, Sibley L, Skinner A (2018). Association of Physical Activity with Income, Race/Ethnicity, and sex among adolescents and young adults in the United States: findings from the National Health and Nutrition Examination Survey, 2007–2016. JAMA Pediatr.

[46] Hamad R, Penko J, Kazi DS, Coxson P, Guzman D, Wei PC (2020). Association of Low Socioeconomic Status with premature Coronary Heart Disease in US adults. JAMA Cardiol.

[47] Kornides ML, Gillman MW, Rosner B, Rimm EB, Chavarro JE, Field AE (2018). U.S. adolescents at risk for not meeting physical activity recommendations by season. Pediatr Res.

[48] Fang K, Mu M, Liu K, He Y (2019). Screen time and childhood overweight/obesity: a systematic review and meta-analysis. Child Care Health Dev.

[49] Saunders TJ, Chaput J-P, Tremblay MS (2014). Sedentary Behaviour as an emerging risk factor for Cardiometabolic diseases in Children and Youth. Can J Diabetes.

[50] Nagata JM, Iyer P, Chu J, Baker FC, Gabriel KP, Garber AK (2021). Contemporary screen time usage among children 9–10-years-old is associated with higher body mass index percentile at 1-year follow-up: a prospective cohort study. Pediatr Obes.

[51] Nagata JM, Trompeter N, Singh G, Ganson KT, Testa A, Jackson DB (2022). Social epidemiology of early adolescent cyberbullying in the United States. Acad Pediatr.

[52] Evans EW, Abrantes AM, Chen E, Jelalian E (2017). Using Novel Technology within a School-based setting to increase physical activity: a pilot study in School-Age children from a Low-Income, Urban Community. Biomed Res Int.

[53] Schneider M, Chau L (2016). Validation of the Fitbit zip for monitoring physical activity among free-living adolescents. BMC Res Notes.

[54] Sundström J, Neovius M, Tynelius P, Rasmussen F (2011). Association of blood pressure in late adolescence with subsequent mortality: cohort study of Swedish male conscripts. BMJ.

[55] Franklin SS, Larson MG, Khan SA, Wong ND, Leip EP, Kannel WB (2001). Does the relation of blood pressure to coronary heart disease risk change with aging? The Framingham Heart Study. Circulation.

[56] Ekelund U, Luan J, Sherar LB, Esliger DW, Griew P, Cooper A (2012). Moderate to vigorous physical activity and sedentary time and cardiometabolic risk factors in children and adolescents. JAMA.

[57] Majid HA, Amiri M, Mohd Azmi N, Su TT, Jalaludin MY, Al-Sadat N (2016). Physical activity, body composition and lipids changes in adolescents: analysis from the MyHeART Study. Sci Rep.

[58] Nagata JM, Vittinghoff E, Gabriel KP, Garber AK, Moran AE, Rana JS, et al. Moderate-to-vigorous intensity physical activity from young adulthood to middle age and metabolic disease: a 30-year population-based cohort study. Br J Sports Med. 2021;56:847–53. 10.1136/bjsports-2021-104231 PMC9017156 34521685

[59] Crowe M, Sampasa-Kanyinga H, Saunders TJ, Hamilton HA, Benchimol EI, Chaput J-P (2020). Combinations of physical activity and screen time recommendations and their association with overweight/obesity in adolescents. Can J Public Health.

