# Supplementary material for: Association of physical activity and screen time with cardiovascular disease risk in the Adolescent Brain Cognitive Development Study
Source: BMC Public Health. 2024 May 18;24:1346. doi: 10.1186/s12889-024-18790-6 (PMC11102349; doi:10.1186/s12889-024-18790-6)
Supplement: Supplementary file 1 — Additional File 1: Appendix A. Flow diagram of included participants. Appendix B. Comparison of participants included vs excluded due to missing data; Appendix C. Associations between screen time and step count categories and binary cardiovascular disease risk (CVD) outcomes in the Adolescent Brain Cognitive Development (ABCD) Study [file 12889_2024_18790_MOESM1_ESM.docx]

Appendix A. Flow chart of study selection based on the inclusion and exclusion criteria.

11,875 participants in from baseline (Year 0, 2016-2018) of the Adolescent Brain Cognitive Development (ABCD) Study

7,140 excluded for missing all CVD risk measurements at Year 2 and Year 3

4,741 Participants included with at least 1 CVD risk measurement at Year 2 or 3

23 excluded for also missing screen time and step count data at Year 2

4,718 Participants included with at least 1 screen time or step count measurement

| Appendix B. Comparison of participants included vs excluded due to missing data. | | | |
| --- | --- | --- | --- |
| Sociodemographic characteristics | Included  (n= 4,718) | Excluded  (n= 7,157) | p |
| Sex |  |  | 0.5931 |
| Female | 47.6% | 48.1% |  |
| Male | 52.4% | 51.9% |  |
| Race/ethnicity |  |  | **0.022** |
| White | 55.3% | 50.1% |  |
| Latino / Hispanic | 15.4% | 18.3% |  |
| Black | 18.1% | 21.6% |  |
| Asian | 5.7% | 6.2% |  |
| Native American | 4.4% | 2.9% |  |
| Other | 1.2% | 0.9% |  |
| Household income |  |  | **0.0279** |
| Less than $75,000 | 36.5% | 38.7% |  |
| $75,000 or more | 63.5% | 61.3% |  |
| Parent education |  |  | 0.0836 |
| High school education or less | 12.9% | 14.0% |  |
| Some college education or more | 87.2% | 86.0% |  |
| Parent marital status |  |  | **0.027** |
| Parent married/partnered | 75.7% | 73.8% |  |
| Parent not married/unpartnered | 24.3% | 26.2% |  |

| Appendix C. Associations between screen time and step count categories and binary cardiovascular disease risk (CVD) outcomes in the Adolescent Brain Cognitive Development (ABCD) Study | | | | | |  |
| --- | --- | --- | --- | --- | --- | --- |
|  |  |  |  |  |  |  |
| Screen time (hrs/day) | AOR (95% CI) | p | Steps/day | AOR (95% CI) | p |  |
| Hypertensive-range blood pressures | | | | | |  |
| Low (0-4) | Reference |  | High (>12,000) | Reference |  |  |
| Medium (>4-8) | 1.28 (0.82 to 2.00) | 0.283 | Medium (>6,000-12,000) | **0.66 (0.44 to 1.00)** | **0.047** |  |
| High (>8) | **1.82 (1.16 to 2.86)** | **0.010** | Low (1,000-6,000) | **0.56 (0.29 to 1.08)** | **0.084** |  |
| Testing consistent with diabetes | | | | | |  |
| Low (0-4) | Reference |  | High (>12,000) | Reference |  |  |
| Medium (>4-8) | 0.59 (0.26 to 1.34) | 0.211 | Medium (>6,000-12,000) | 1.68 (0.64 to 4.41) | 0.293 |  |
| High (>8) | **1.93 (1.00 to 3.73)** | **0.051** | Low (1,000-6,000) | 2.20 (0.72 to 6.71) | 0.166 |  |
| High total cholesterol | | | | | |  |
| Low (0-4) | Reference |  | High (>12,000) | Reference |  |  |
| Medium (>4-8) | 1.13 (0.38 to 3.35) | 0.822 | Medium (>6,000-12,000) | 1.80 (0.48 to 6.78) | 0.384 |  |
| High (>8) | 1.33 (0.39 to 4.48) | 0.649 | Low (1,000-6,000) | 0.69 (0.08 to 5.70) | 0.733 |  |
| Low HDL cholesterol | | | | | |  |
| Low (0-4) | Reference |  | High (>12,000) | Reference |  |  |
| Medium (>4-8) | 0.88 (0.39 to 1.99) | 0.759 | Medium (>6,000-12,000) | 1.54 (0.67 to 3.59) | 0.312 |  |
| High (>8) | 1.52 (0.69 to 3.36) | 0.299 | Low (1,000-6,000) | 0.65 (0.12 to 3.47) | 0.612 |  |
| All models include screen time and physical activity (step count) as the joint independent variables and were adjusted for age, sex, race/ethnicity, household income, parental educational level, parental marital status, and data collection period (i.e., before the COVID-19 pandemic, before and during the COVID-19 pandemic, or during the COVID-19 pandemic), and calendar month. For diabetes, high total cholesterol, and low HDL cholesterol, time between independent and dependent variable was also adjusted for given that some measures were collected across Years 2 and 3. Participants with a prior diagnosis of diabetes were excluded from the analysis of diabetes and participants on hypertension medications were excluded from analyses of hypertensive-range blood pressures. | | | | | |  |
|  |  |  |  |  |  |  |
|  |  |  |  |  |  |  |
|  |  |  |  |  |  |  |
|  |  |  |  |  |  |  |
